# Bladder Cancer Chemosensitivity Is Affected by Paraoxonase-2 Expression

**DOI:** 10.3390/antiox9020175

**Published:** 2020-02-20

**Authors:** Stefania Fumarola, Monia Cecati, Davide Sartini, Gianna Ferretti, Giulio Milanese, Andrea Benedetto Galosi, Valentina Pozzi, Roberto Campagna, Camilla Morresi, Monica Emanuelli, Tiziana Bacchetti

**Affiliations:** 1 Department of Clinical Sciences, Polytechnic University of Marche, 60131 Ancona, Italy; stefy.fumarola@gmail.com (S.F.); moniacecati@gmail.com (M.C.); d.sartini@staff.univpm.it (D.S.); g.ferretti@univpm.it (G.F.); g.milanese@univpm.it (G.M.); a.b.galosi@univpm.it (A.B.G.); rob_campagna@yahoo.com (R.C.); 2 New York-Marche Structural Biology Center, Polytechnic University of Marche, 60131 Ancona, Italy; valentinapozzi81@gmail.com; 3 Department of Life and Environmental Sciences, Polytechnic University of Marche, 60131 Ancona, Italyt.bacchetti@staff.univpm.it (T.B.)

**Keywords:** Bladder cancer, paraoxonase-2, chemotherapeutic drugs, cell viability, oxidative stress

## Abstract

The goal of the current study was to identify potential roles of paraoxonase-2 in bladder carcinogenesis. T24 bladder cancer cells were transfected with plasmids inducing paraoxonase-2 silencing or overexpression. Upon the selection of clones stably down- or upregulating paraoxonase-2, cell proliferation, migration, and the production of reactive oxygen species were evaluated, before and after treatment with cisplatin and gemcitabine, used alone or in combination. The activity levels of both caspase-3 and caspase-8 were also analyzed. shRNA-mediated gene silencing and the overexpression of paraoxonase-2 revealed that the enzyme was able to promote both the proliferation and migration of T24 cells. Moreover, the knockdown of paraoxonase-2 was significantly associated with a reduced cell viability of T24 cells treated with chemotherapeutic drugs and led to both an increase of reactive oxygen species production and caspase-3 and caspase-8 activation. Conversely, under treatment with anti-neoplastic compounds, a higher proliferative capacity was found in T24 cells overexpressing paraoxonase-2 compared with controls. In addition, upon enzyme upregulation, both the production of reactive oxygen species and activation of caspase-3 and caspase-8 were reduced. Although further analyses will be required to fully understand the involvement of paraoxonase-2 in bladder tumorigenesis and in mechanisms leading to the development of chemoresistance, the data reported in this study seem to demonstrate that the enzyme could exert a great impact on tumor progression and susceptibility to chemotherapy, thus suggesting paraoxonase-2 as a novel and interesting molecular target for effective bladder cancer treatment.

## 1. Introduction

Bladder cancer (BC) is the ninth most common cancer in the world. The incidence of this neoplasm is gender-related, meaning that its frequency is 3-4 times higher in men than in women [1]. Moreover, BC is more frequent at an advanced age and the greatest number of cases occurs in people over 75 years old [1]. Smoking habits and exposure to some chemical compounds also increase the risk of developing BC [1]. Approximately 25% of patients diagnosed with BC display muscle-invasive disease, which is often associated with the presence or future development of metastasis [2]. Concerning unresectable and metastatic forms, systemic chemotherapy is the standard initial treatment. In particular, cisplatin or cisplatin-gemcitabine regimens have been the standard of care for many years [3]. In particular, cisplatin-based chemotherapy is considered standard first-line treatment for patients with metastatic BC [4]. The initial response rates resulting from this chemotherapeutic treatment are high, but the median survival is approximately 15 months [5,6]. Unfortunately, this is mainly due to the fact that BC cells either intrinsically are or rapidly become resistant to cisplatin, leading to therapeutic failure and relapse. One of the major clinically relevant issues is that cisplatin resistance often exhibits a multifactorial nature [7].

Cisplatin becomes active upon entering the cell. Once in the nucleus, it binds to the purine N7 atom, triggering DNA damage in cancer cells, blocking cell division, and resulting in apoptotic cell death. Moreover, oxidative stress is another key mechanism involved in cisplatin toxicity. Mitochondria are the major target for the oxidative stress that is cisplatin-induced, resulting in the loss of the mitochondrial protein sulfhydryl group, preventing calcium uptake and triggering a decrease of the mitochondrial membrane potential [8]. The copper transporter Ctr1 mediates cisplatin uptake by the cells and the genetic knock-out of CTR1 was demonstrated to induce cisplatin resistance in vivo. Therefore, cells with an increased CTR1 expression display an increased sensitivity to cisplatin [9]. Moreover, the gene TMEM205, encoding for a membrane protein, was demonstrated to be involved in the cellular resistance to cisplatin [10].

In combination with cisplatin, gemcitabine is also used for the treatment of BC. Once in the cytoplasm, gemcitabine is phosphorylated by deoxycytidine kinase (dCK). In turn, this compound is further converted by other pyrimidine kinases to its active diphosphate and triphosphate derivatives [11]. These derivatives exert their toxic effect through different mechanisms, which are all based on DNA damage. In its DNA-incorporated form, gemcitabine causes DNA polymerase dislodgement one nucleotide downstream of the gemcitabine itself. Given the presence of one nucleotide downstream of the gemcitabine, the break site is not attractive for the DNA repair enzymes responsible for the excision of mismatched deoxynucleotides from the DNA [12]. Additionally, gemcitabine induces the production of reactive oxygen species (ROS), which are responsible for DNA damage [13]. Due to the central role of dCK in gemcitabine metabolism, its deficiency is a major contributor to gemcitabine resistance [14,15]. Moreover, the excision repair cross-complementation 1 (ERCC1), which can repair strand breaks, was shown to be overexpressed in poor gemcitabine responders [16,17]. 

To improve the outcome of clinical N0M0 patients with muscle-invasive BC, the addition of chemotherapy (neoadjuvant before radical cystectomy or adjuvant after surgery) is used [18]. The advantages of neoadjuvant chemotherapy are the presumed low burden of micrometastatic disease, the ability to test the chemosensitivity by following the primary tumor, and the expected better tolerability of chemotherapy before rather than after surgery. A disadvantage is the delay of surgery in non-responders. However, neoadjuvant chemotherapy does not seem to have an impact on surgery [19]. The latest and largest meta-analysis published in 2005 indicated a significant 5% absolute survival benefit in favor of neoadjuvant chemotherapy [20].

Adjuvant chemotherapy is recommended for high-risk M0 BC patients. The advantage of adjuvant chemotherapy is that the treatment of low-risk patients can be avoided because the pathologic stage is known, whereas the disadvantages are the delay of chemotherapy, worse tolerability, lower likelihood of receiving it after surgery, and absence of measurable disease. In a meta-analysis, 945 patients were included from nine trials, but none of the included trials were significantly positive for overall survival in favor of adjuvant chemotherapy. However, the disease-free survival benefit was more apparent in studies with higher nodal involvement (Hazard Ratio: 0.39; 95% Confidence Interval, 0.28–0.54) [21].

In this work, attention was focused on the enzyme paraoxonase-2 (PON2). PON2 is an intracellular protein with a relative molecular mass of approximately 44 kDa [22]. In humans, PON2 is encoded by a gene belonging to the paraoxonase family, which also includes those for paraoxonase-1 and paraoxonase-3. *PON2* is widely expressed in many cellular types and tissues, including vascular cells [22]. Upon translation, protein is incorporated into the lipid bilayer thanks to its transmembrane domain. PON2 is located in the cell plasma membrane, the endoplasmic reticulum (ER), and mitochondria. Nevertheless, data on the predominant distribution of PON2 inside the cell are rather controversial because of its dynamic translocation from the cytosol to the plasma membrane in response to oxidative stress. Oxidative stress is responsible for intracellular calcium release and subsequent peroxidation of the lipid bilayer at the plasma membrane. 

Hagmann et al. demonstrated that the calcium signal triggers the translocation of PON2 to the plasma membrane [23]. The main function exerted by the enzyme within cells is mainly related to its antioxidant activity. Indeed, Ng et al. demonstrated that PON2 contributes, together with other intracellular enzymes and systems, to protecting cells from oxidative stress [24]. Due to its intracellular localization, as well as its antioxidant function, PON2 was reported to display an anti-apoptotic role, with potential consequences for tumor cell behavior [25]. Over the past years, many studies have described the involvement of PON2 in cancer. In particular, *PON2* expression was shown to be increased in some solid tumors, including pancreatic cancer [26], glioblastoma multiforme [27], and recently BC [28]. Concerning bladder cancer, in our previous study, we demonstrated that the enzyme levels were significantly higher in tumors compared with adjacent normal looking tissue samples from BC patients. Moreover, preliminary results obtained from analyses performed on bladder cancer cell lines seemed to suggest that PON2 is able to promote cell proliferation and resistance to oxidative stress [28].

The analyses carried out in the present study aimed to further investigate the role of PON2 in BC. Enzyme silencing and overexpression were induced in the T24 bladder cancer cell line. Subsequently, T24 cell proliferation, migration, and susceptibility to oxidative stress were evaluated, before and after treatment with cisplatin and gemcitabine. In addition, the activity levels of both caspase-3 and caspase-8, as key regulators of the apoptotic response, were investigated. 

## 2. Materials and Methods 

### 2.1. Cell Lines and Culture Conditions

The human bladder cancer cell line T24, obtained from the American Type Culture Collection (ATCC, Rockville, MD, USA), was maintained in DMEM/F12 medium, as previously described [28].

### 2.2. Cloning

The plasmid vector pLKO.1-647 containing stem-loop cassette encoding short hairpin RNA (shRNA) targeted to human *PON2* (Sigma-Aldrich, St. Louis, MO, USA) was used for PON2 gene silencing. For the induction of *PON2* overexpression, the plasmid construct pcDNA3-PON2 was obtained as described elsewhere [28].

### 2.3. Transfection

To achieve *PON2* silencing, T24 cells were seeded in 24-well plates (4 × 10^4^ cells/well) the day before transfection. The plasmids against *PON2* (pLKO.1-647) or the empty vector (pLKO.1-puro) were used to transfect 80% confluent cells (0.5 µg/well). Control cells were treated with transfection reagent only (mock).

To induce the overexpression of PON2, cells were seeded in 6-well plates (2.4 x 10^5^ cells/well) the day before transfection and were then transfected with the pcDNA3-PON2 plasmid vector (3μg/well). Control cells were transfected with the empty vector (pcDNA3) or treated with transfection reagent only (mock).

Both procedures were performed using FuGENE HD Transfection Reagent (Promega, Madison, WI, USA), following the manufacturer’s instructions. Forty-eight hours from the beginning of the transfection, culture medium was discarded and replaced with complete medium containing puromycin (1 μg/mL) or geneticin (800 μg/mL), in order to select cellular clones downregulating or overexpressing PON2, respectively. For all subsequent experiments, puromycin- and geneticin-resistant cells were maintained in complete selection medium. The efficiency of PON2 silencing and overexpression in T24 cells were evaluated by Real-Time PCR and Western blot analysis.

### 2.4. Real-Time PCR

Quantitative Real-Time PCR was performed as reported elsewhere [28]. The relative expression of *PON2* was calculated by the 2^−ΔΔCt^ method. Each experiment was performed in triplicate and independently repeated three times. 

### 2.5. Western Blot Analysis 

A Western blot assay was set up to evaluate PON2 protein levels, as previously reported [28]. Each experiment was performed in triplicate and independently repeated three times.

### 2.6. Monolayer Wound Healing Assay

To evaluate the migration capacity, T24 cells were seeded in 6-well plates (6 × 10 ^5^ cells/well) and allowed to attach and grow up to 90–100% confluency. Cell monolayers were scratched using a sterile 200 μL pipette tip to make a vertical wound. Wounded monolayers were then washed three times to remove cell debris and incubated in medium containing 0.5% FBS (Fetal Bovine Serum). Upon medium replacement, cells were monitored under a microscope equipped with a camera (Deiss) and photographed at 6, 12, and 24 h. Each experiment was performed in triplicate and independently repeated three times.

### 2.7. Chemotherapeutic Treatment

T24 cells downregulating and overexpressing *PON2*, as well as controls, were seeded in 96-well plates (3 × 10^3^ cells/well). The day after seeding, cells were treated with cisplatin and gemcitabine, as previously reported [29,30]. In particular, cells were incubated with cisplatin (20 μmol/L), gemcitabine (6.25 μmol/L), or a combination of both drugs (1.0 μmol/L cisplatin and 1.56 μmol/L gemcitabine) for 24 h. After incubation with drugs, cells were washed with phosphate-buffered saline and complete fresh medium was added. Cell viability and susceptibility to oxidative stress were further evaluated using 3-(4,5-dimethylthiazol-2-yl)-2,5-diphenyltetrazolium bromide (MTT) assays and a 2’,7’-dichlorodihydrofluorescein diacetate (H_2_DCF-DA) probe, respectively. 

### 2.8. MTT Assay

Cell proliferation was determined using a colorimetric assay with 3-(4,5-dimethylthiazol-2-yl)-2,5-diphenyl tetrazolium bromide (MTT), as described elsewhere [28]. Cell viability was evaluated in untreated cells (0 h) and at different time points (12, 24, 36, 48, and 72 h) after starting the treatment with drugs. Each experiment was performed in triplicate and independently repeated three times.

### 2.9. Detection of Intracellular Oxidative Stress

To evaluate the intracellular oxidative stress, the oxidation of H_2_DCF-DA (Sigma-Aldrich, St. Louis, MO, USA) was measured as previously reported [31]. ROS production was evaluated in untreated cells (0 h) and at different time points (12, 24, 36, 48, and 72 h) after starting the treatment with drugs. Each experiment was performed in triplicate and independently repeated three times.

### 2.10. Determination of Caspase-3 and Caspase- 8 Activity

Caspase-3 and caspase-8 activities were determined, using the Caspase-3/CPP32 and Caspase-8/FLICE Colorimetric Assay Kits (Biovision, Milpitas, CA, USA), respectively, in cells after 24h treatment with chemotherapeutic drugs or with 1µM Dexamethasone (positive control). The assay was run following the manufacturer’s instructions.

Each experiment was performed in triplicate and independently repeated three times.

### 2.11. Statistical Analysis

Data were analysed using GraphPad Prism software version 8.00 for Windows (GraphPad Software, San Diego, CA, USA). Differences between groups were determined using one-way analysis of variance (ANOVA). A *p*-value < 0.05 was accepted as statistically significant.

## 3. Results

### 3.1. Efficiency of PON2 shRNA-Mediated Knockdown and Overexpression in T24 Cells

T24 cells were transfected as reported in the materials and methods section. To evaluate the efficiency of transfection, PON2 mRNA and protein levels were evaluated by Real-Time PCR and Western blot analysis, respectively.

**Figure 1:**
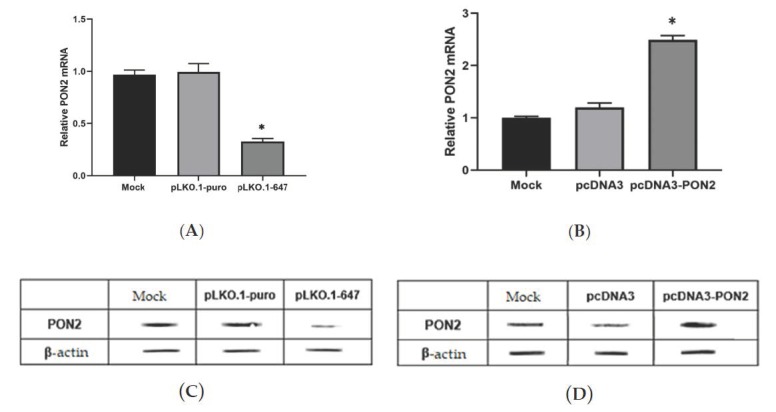
Paraoxonase-2 (*PON2*) expression levels in T24 cells. In order to induce *PON2* silencing or overexpression, T24 cells were transfected with pLKO.1-647 or pcDNA3-PON2, respectively. Control cells were treated with empty vectors (pLKO.1-puro or pcDNA3) or with transfection reagent only (mock). Real-Time PCR was used to evaluate *PON2* mRNA levels in cells transfected with plasmids inducing enzyme down- (panel **A**) or upregulation (panel **B**) compared with the mock. Lysates obtained from T24 cells were analyzed by Western blot to evaluate PON2 protein levels upon enzyme knockdown (panel **C**) or overexpression (panel **D**). All values reported in panels A and B are expressed as the mean ± standard deviation (*p < 0.05).

Compared with the mock and empty vector, *PON2* expression levels were significantly reduced upon treatment with pLKO.1–647. Real-Time PCR showed a significant (*p* < 0.05) downregulation of *PON2* in cells transfected with the pLKO.1–647 plasmid (0.32 ± 0.05) compared with the mock (1.00 ± 0.09) and those transfected with the empty vector (0.99 ± 0.08) (Figure 1A). On the contrary, T24 cells transfected with pcDNA3-PON2 displayed a significant (*p* < 0.05) enzyme upregulation (2.49 ± 0.09) with respect to cells treated with pcDNA3 (1.20 ± 0.08) and the mock (1.00 ± 0.07) (Figure 1B).

PON2 expression was also detected at the protein level by Western blot analysis. The results obtained showed that, compared with controls, cells transfected with pLKO.1–647 or pcDNA3-PON2 plasmid vectors displayed markedly decreased or increased PON2 levels, respectively (Figure 1C,D).

### 3.2. Effect of PON2 Knockdown and Overexpression on T24 Cell Proliferation and Migration 

To examine the role of PON2 in tumor cell metabolism and analyze the biological effect associated with enzyme knockdown or overexpression, the cell viability and migration capacity were analyzed at different time points in T24 cells.

**Figure 2:**
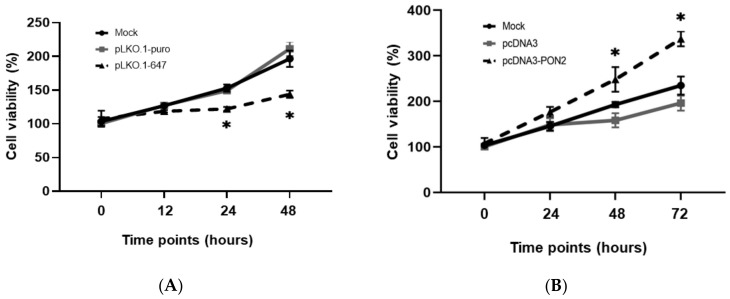
Evaluation of T24 cell viability. The effect of *PON2* silencing (panel **A**) and overexpression (panel **B**) on cell viability was assessed by the MTT assay. Cell viability was evaluated in the mock and cells treated with plasmids at different time points, between 0 and 72 h. All values are expressed as the mean ± standard deviation (*p < 0.05).

The effect of *PON2* knockdown and overexpression on cell proliferation was evaluated by the MTT assay. As shown in Figure 2A, enzyme knockdown led to a significant (*p* < 0.05) decrease in cell growth of T24 cells starting from the 24 h time-point with respect to control cells (mock), as well as those treated with an empty vector (pLKO.1-puro). On the other side, enzyme upregulation was associated with a significant (*p* < 0.05) increase in cell proliferation of T24 cells starting from the 48h time-point (Figure 2B).

**Figure 3:**
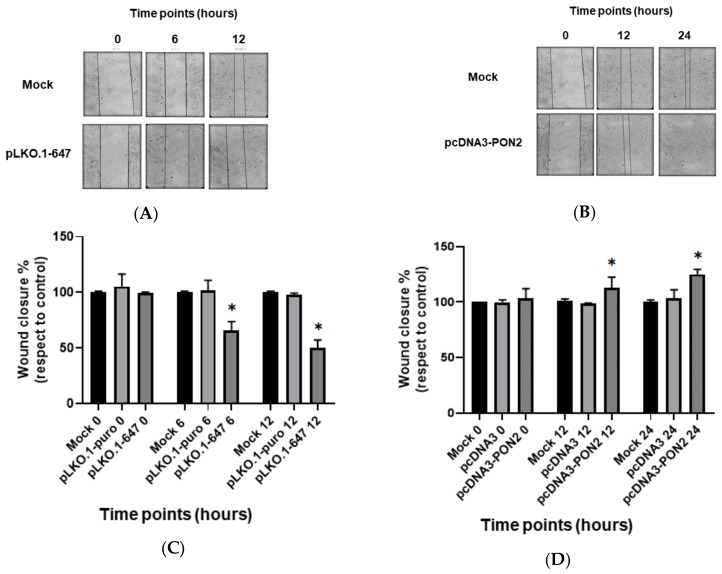
Migration ability of T24 cells. Wound healing assays were used to assess the impact of *PON2* expression on cell migration. T24 cells transfected with the plasmid aimed to induce enzyme knockdown (panel **A**) or overexpression (panel **B**) were photographed immediately (0 h) after wounding by a pipette tip and at different time points, ranging between 6 and 24 h. The migration ability of T24 cells down- (panel **C**) or upregulating *PON2* (panel **D**) was evaluated by measuring their efficiency in wound repair compared with that of mock samples. All values are expressed as the mean ± standard deviation (*p < 0.05).

To assess the biological influence of PON2 on cell migration, T24 cells transfected with pLKO.1–647 or pcDNA3-PON2 plasmids were subjected to a wound healing assay. Compared with the mock-treated cells, the migration ability of T24 cells significantly (*p* < 0.05) decreased at 6h (32% reduction) and 12 h (50% reduction) time-points after *PON2* silencing (Figure 3A–C). On the contrary, the overexpression of *PON2* led to a promotion of the migration ability (*p* < 0.05) of T24 cells at 12 h (13% increase) and 24h (25% increase) time-points with respect to the control (Figure 3B–D).

### 3.3. PON2 Influence on the Sensitivity of T24 Cells to Treatment with Chemotherapeutic Drugs

**Figure 4:**
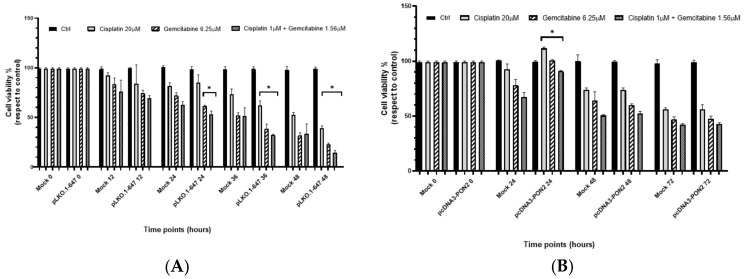
Effect of chemotherapeutic drugs on the proliferation of T24 cells. The MTT assay was performed to evaluate the influence of treatment with cisplatin (20 µM), gemcitabine (6.25 µM), or both compounds (cisplatin 1 µM + gemcitabine 1.56 µM) on the viability of cells transfected with pLKO.1-647 (panel **A**) or pcDNA3-PON2 (panel **B**) compared with controls (mock). Determination of the proliferative rate was carried out at different time points (0, 12, 24, 36, 48, and 72 h). All values are expressed as the mean ± standard deviation (*p < 0.05).

The MTT colorimetric assay was used to evaluate the effect of chemotherapeutic drugs administrated as reported in the materials and methods section on cell viability. In agreement with previously reported results [29], chemotherapeutic drugs significantly reduced the cell viability of T24 control cells (Figure 4).

As demonstrated in Figure 4A, treatment with cisplatin alone led to a significant (*p* < 0.05) decrease in the proliferative capacity of cells transfected with pLKO.1-647 compared with that of the mock, after 36h (15% reduction) and 48h (25% reduction). Treatment with gemcitabine alone or with both drugs was significantly (*p* < 0.05) associated with a reduction in the viability of *PON2* downregulated cells (pLKO.1-647) with respect to that of the mock at 24 h (14% reduction for gemcitabine and 16% reduction for both drugs), 36 h (27% reduction for gemcitabine and 37% reduction for both drugs), and 48 h (28% reduction for gemcitabine and 59% reduction for both drugs) time points (Figure 4A).

Conversely, the induction of *PON2* overexpression led to a significant (*p* < 0.05) enhancement in the proliferative capacity of T24 cells (pcDNA3-PON2) compared with controls (mock) at the 24 h time-point (21% increase for cisplatin, 28% increase for gemcitabine, and 36% reduction for both drugs). However, this effect seemed to be lost at higher time points (Figure 4B).

### 3.4. Effect of PON2 Expression on the ROS Production of T24 Cells Treated with Chemotherapeutic Drugs

**Figure 5:**
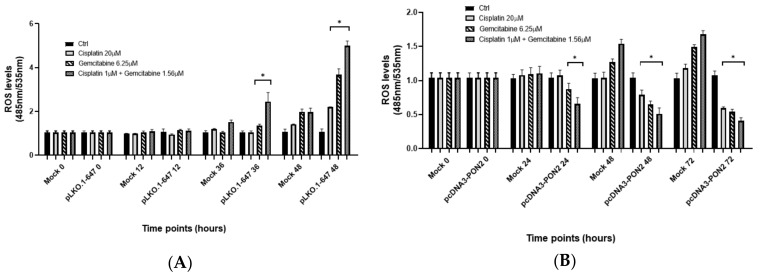
Intracellular Reactive Oxygen Species (ROS) levels in T24 cells upon treatment with drugs. In vitro effect of *PON2* silencing (panel **A**) and overexpression (panel **B**) on intracellular ROS production upon treatment with cisplatin (20 µM), gemcitabine (6.25 µM), or both compounds (cisplatin 1 µM + gemcitabine 1.56 µM). ROS levels were determined at different time points (0, 12, 24, 36, 48, and 72 h). All values are expressed as the mean ± standard deviation (*p < 0.05).

To assess the effect of the induction of *PON2* knockdown and overexpression on oxidative stress, intracellular Reactive Oxygen Species (ROS) levels were evaluated in T24 cells after incubation with chemotherapeutic drugs, as well as in untreated cells. Chemotherapeutic treatment led to a significant increase of ROS production in T24 control cells (Figure 5).

As shown in Figure 5A, upon treatment with cisplatin, ROS production was significantly (*p* < 0.05) higher in *PON2* downregulated cells (pLKO.1-647) compared with mock-treated cells at the 48h time point only (1.56-fold increase). Incubation with gemcitabine alone or with the cisplatin/gemcitabine combination led to significantly (*p* < 0.05) higher ROS levels in T24 cells transfected with pLKO.1-647 compared with the mock, both at 36h (1.30-fold increase for gemcitabine and 1.61-fold increase for both drugs) and 48h (1.85-fold increase for gemcitabine and 2.55-fold increase for both drugs) time points (Figure 5A).

On the contrary, upon treatment with cisplatin and gemcitabine, used alone or in combination, intracellular ROS production was significantly (*p* < 0.05) lower in *PON2* overexpressing cells compared with the mock at 48h (1.37-fold decrease for cisplatin, 1.96-fold decrease for gemcitabine, and 3.03-fold decrease for both drugs) and 72 h (2.00-fold decrease for cisplatin, 2.78-fold decrease for gemcitabine, and 4.00-fold decrease for both drugs) time points, while cisplatin alone had no significant effect at 24 h (1.26-fold decrease for gemcitabine and 1.69-fold decrease for both drugs) (Figure 5B). 

### 3.5. PON2 Protects T24 Cells against Apoptosis through Caspase-3 and Caspase-8 Activation

In order to investigate the contribution of PON2 to BC tumorigenesis by promoting apoptotic escape, caspase-3 and caspase-8 activities were evaluated in T24 cells expressing either reduced or elevated *PON2* levels, as well as corresponding controls, upon treatment for 24 h with chemotherapeutic drugs.

**Figure 6:**
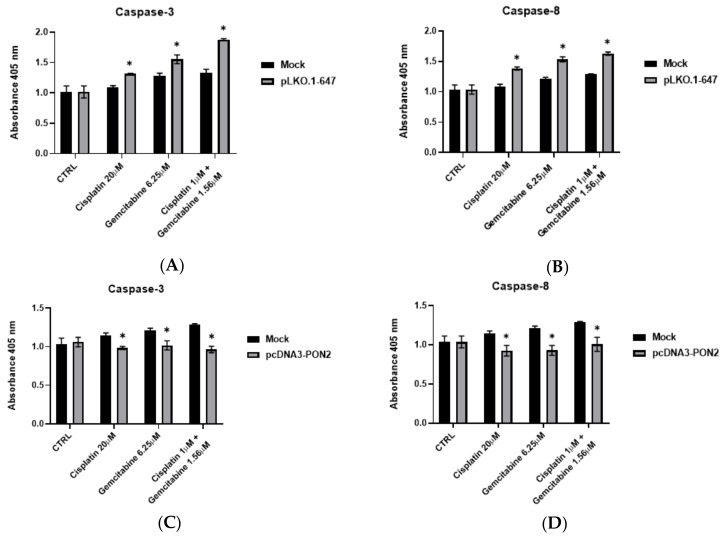
Caspase-3 and caspase-8 activity in T24 cells treated with chemotherapeutic compounds. Apoptosis induction in *PON2* downregulated (panels **A** and **B**) and overexpressing (panels **C** and **D**) cells was determined by evaluating the activity levels of both caspase-3 and caspase-8 after 24 h treatment with cisplatin (20 µM), gemcitabine (6.25 µM), or both compounds (cisplatin 1 µM + gemcitabine 1.56 µM). All values are expressed as the mean ± standard deviation (*p < 0.05).

Treatment was able to induce a significant (*p* < 0.05) activation of both caspase-3 and caspase-8 in T24 cells transfected with pLKO.1-647 compared with the mock. In particular, caspase-3 activation was higher in *PON2* downregulated cells treated with both compounds (1.41-fold increase) than in T24 cells upon incubation with cisplatin (1.20-fold increase) or gemcitabine (1.21-fold increase) used alone. On the other hand, the activation of caspase-8 did not seem to be significantly different (pLKO.1-647 versus mock) among treatments (1.28-fold increase for cisplatin, 1.26-fold increase for gemcitabine, and 1.23-fold increase for both drugs) (Figure 6A,B).

On the other hand, T24 cells overexpressing *PON2* showed significantly (*p* < 0.05) lower levels of caspase-3 and caspase-8 activity than those determined in the mock. Concerning caspase-3, the reduction of activity levels was enhanced in *PON2* overexpressing cells that underwent combined treatment (1.35-fold reduction) compared with that observed after incubation with cisplatin (1.18-fold reduction) or gemcitabine (1.19-fold reduction) alone. Conversely, the decrease of caspase-8 activity detected in T24 cells upon enzyme overexpression (pcDNA3-PON2) did not seem to be significantly altered based on treatment with different compounds or the drug combination (1.25-fold reduction for cisplatin, 1.30-fold decrease for gemcitabine, and 1.28-fold decrease for both drugs) (Figure 6C,D).

## 4. Discussion

In recent years, great efforts have been made to identify molecules that are involved in BC tumorigenesis and/or are able to reflect changes in neoplastic tissue, thus acting as potential biomarkers for early and noninvasive BC detection [32]. Moreover, chemoresistance remains a critical problem in patients with BC due to the lack of effective second-line therapies, and therefore, a better understanding of chemoresistance mechanisms in BC is urgently required [33].

The known mechanisms by which BC becomes resistant to platinum drugs are multifactorial, including: (1) Reduced intracellular drug uptake consequent to a downregulation of CTR1; (2) promoted drug efflux by increasing cellular glutathione (GSH), as shown by Kotoh et al., who demonstrated that the content of GSH was significantly increased in the cisplatin-resistant BC cell line (T24/DDP7), compared with the level of GSH in the T24 parental line; 3) enhanced DNA damage repair through the increased activity of nucleotide excision repair (NER) and homologous recombination repair (HR); 4) impairments of the apoptotic pathways, such as the loss of expression of p53 [34,35].

However, chemotherapeutic drugs can increase ROS levels, and most cancer cells treated with chemotherapeutics suffer from ROS-mediated apoptosis [36]. 

Some cancer cells can evolve and develop mechanisms to escape ROS-mediated apoptosis and, thus, acquire tolerance to anti-cancer drugs [37]. The ROS system has a dual function that can either induce apoptosis or allow cells to adapt to various environments; thus ROS regulation has been proposed as a critical target for developing anticancer drugs [38].

There is a growing scientific consensus that recognizes a possible role of PON2 in the physiopathology of cancer. In fact, several studies have reported the upregulation of *PON2* in different human cancers [25,39], including BC [28].

In this work, the potential role of PON2 in the in vitro tumorigenicity of BC cells was investigated. *PON2* knockdown and overexpression were induced in T24 human bladder cancer cells further treated with chemotherapeutic drugs. Subsequent analyses were carried out to evaluate the effect of the modulation of *PON2* expression on cell proliferation, migration, response to oxidative stress, and apoptosis.

The reported data demonstrated that *PON2* expression exerts a positive influence on T24 cell proliferation and migration, thus highlighting its potential role in promoting bladder tumorigenesis. In particular, *PON2* overexpression led to an increase in the cell viability of T24 cells treated with chemotherapeutic drugs, while *PON2* downregulation was associated with a significant reduction of their proliferative capacity. As reported in the literature, these results confirmed that PON2 is involved in the survival and proliferation of cancer cells [40].

Moreover, ROS production, known to be induced upon treatment with cisplatin and gemcitabine [31,41], was significantly affected by *PON2* dysregulation. Indeed, *PON2* downregulation led to an increase of ROS levels in T24 bladder cancer cells treated with chemotherapeutic agents. On the contrary, *PON2* upregulation significantly counteracted the increase in cellular ROS production in response to oxidative stress triggered by chemotherapeutic compounds. This evidence is in accordance with data presented in the literature showing that PON2 enzyme activity protected macrophages, vascular, and other cells against oxidative stress, whereas *PON2* downregulation reversed these effects [24,42].

The results obtained from further in vitro experiments demonstrated the influence of *PON2* expression on drug-induced caspase activation. After treatment with chemotherapeutic drugs, we observed a comparative increase in caspase activity upon *PON2* silencing in T24 cells, and a reduction in *PON2*-overexpressing cells.

Our results are consistent with data presented in the literature coming from other studies which have investigated the involvement of PON2 in cancer. In particular, the role played by PON2 as a metabolic regulator has been confirmed in a study carried out by Nagarajan et al. In pancreatic ductal adenocarcinoma (PDAC) cell lines, the authors demonstrated that PON2 transcriptional repression is responsible for affecting the transport activity of glucose transporter 1 and subsequently, for an inhibition of PDAC tumor growth and metastasis [25]. Similarly, Tseng et al. demonstrated that valproic acid, decreasing the PON2 expression, made glioblastoma multiforme-derived cell lines more sensitive to oxidative damage and cell death [27].

It is well-known that cancer cell responses to apoptotic insults are significantly affected by the cellular redox status. On the other hand, it is also well-established that oxidative stress is closely linked to cell death and cancer [43,44,45]. The anti-apoptotic effect exerted by PON2 is reasonably strictly related to its intracellular localization, mainly within the membranous systems of the ER and mitochondria. Altenhofer et al. recognized the ability of PON2 to prevent mitochondria-derived superoxide formation, which is responsible for the activation of the apoptotic pathway in response to cardiolipin peroxidation and subsequent cytochrome c release [46].

Many studies have reported results that have facilitated understanding of the anti-apoptotic roles of PON2. The activation of executioner caspase-3 and subsequent induction of apoptosis can arise from longer-lasting ER stress, which triggers the unfolded protein response pathway [47]. Horke et al. demonstrated that human umbilical vein endothelial cells EA.hy 926, stably overexpressing *PON2*, displayed a significant reduction of caspase 3/7 activation, although treated with the unfolded protein response (UPR)-inducing reagent tunicamycin. Conversely, in *PON2*-silenced EA.hy 926 cells, treatment with tunicamycin led to an opposite effect and an increase of the caspase 3/7 activation status [26]. Further results demonstrated that PON2 could confer protection against ER-stress-induced caspase-3 activation, by the modulation of calcium homeostasis [48].

The data reported in the present in vitro study demonstrate that PON2 contributes to bladder tumorigenesis by promoting both cell proliferation and migration. Moreover, the enzyme was found to play a significant role in BC cell resistance to chemotherapeutic treatment with cisplatin and gemcitabine.

## 5. Conclusions

In this light, *PON2* overexpression in BC could represent an adaptive mechanism of tumor cells to escape/survive cell death and apoptosis induced by chemotherapy.

Although further analyses will be required to deeply investigate the molecular mechanisms by which PON2 could participate in bladder carcinogenesis, our study clearly demonstrates, for the first time, the impact of the enzyme on tumor progression and the susceptibility of tumor cells to chemotherapeutics, thus suggesting a potential use of *PON2* as an interesting molecular target for BC therapy. Further studies are required to solve the PON2 protein structure in order to develop selective inhibitors of the enzyme, which could be combined with chemotherapeutic drugs to improve the BC outcome. However, given the wide expression of PON2, it is necessary to design a proper drug-delivery strategy in order to selectively address these molecules to the BC cells.
